# Human development, social vulnerability and COVID-19 in Brazil: a study of the social determinants of health

**DOI:** 10.1186/s40249-020-00743-x

**Published:** 2020-08-31

**Authors:** Carlos Dornels Freire de Souza, Michael Ferreira Machado, Rodrigo Feliciano do Carmo

**Affiliations:** 1 Department of Medicine, Post-Graduation Program in Health Family, Federal University of Alagoas (UFAL), Campus Arapiraca. Rodovia AL-115, Bom Sucesso, Arapiraca, Alagoas 57309-005 Brazil; 2 Postgraduate Program in Health and Biological Sciences, Federal University of Vale do São Francisco (UNIVASF), Petrolina, Brazil; 3 Postgraduate Program in Biosciences, Federal University of Vale do São Francisco (UNIVASF), Petrolina, Brazil

**Keywords:** Coronavirus, Ecological study, Human development, Social vulnerability

## Abstract

**Background:**

Coronavirus disease 2019 (COVID-19) was confirmed in Brazil in February 2020. Since then, the disease has spread throughout the country, reaching the poorest areas. This study analyzes the relationship between COVID-19 and the population’s living conditions. We aimed to identify social determinants related to the incidence, mortality, and case fatality rate of COVID-19 in Brazil, in 2020.

**Methods:**

This is an ecological study evaluating the relationship between COVID-19 incidence, mortality, and case fatality rates and 49 social indicators of human development and social vulnerability. For the analysis, bivariate spatial correlation and multivariate and spatial regression models (spatial lag model and spatial error models) were used, considering a 95% confidence interval and a significance level of 5%.

**Results:**

A total of 44.8% of municipalities registered confirmed cases of COVID-19 and 14.7% had deaths. We observed that 56.2% of municipalities with confirmed cases had very low human development (COVID-19 incidence rate: 59.00/100 000; mortality rate: 36.75/1 000 000), and 52.8% had very high vulnerability (COVID-19 incidence rate: 41.68/100 000; mortality rate: 27.46/1 000 000). The regression model showed 17 indicators associated with transmission of COVID-19 in Brazil.

**Conclusions:**

Although COVID-19 first arrived in the most developed and least vulnerable municipalities in Brazil, it has already reached locations that are farther from large urban centers, whose populations are exposed to a context of intense social vulnerability. Based on these findings, it is necessary to adopt measures that take local social aspects into account in order to contain the pandemic.

## Background

In January 2020, a new coronavirus called severe acute respiratory syndrome coronavirus 2 (SARS-CoV-2) was identified, causing coronavirus disease 2019 (COVID-19) [1]. On March 11, 2020 the WHO declared a pandemic [2].

As of April 11, 2020, there were more than 4.1 million cases and 284 000 deaths due to the disease worldwide [3]. The countries with the highest number of cases are the USA (1 300 000), Spain (224 000), and the United Kingdom (224 000). In number of deaths, the USA (79 000), the United Kingdom (32 000), and Italy (30 000) have the highest numbers [3].

In Brazil, the first case of COVID-19 was confirmed on February 26, 2020, and the first death was confirmed on March 17, in São Paulo, the country’s most populous metropolis, with approximately 12 million inhabitants [4]. The disease spread rapidly to other Brazilian states. On April 11, 2020 the country had 163 000 cases and 11 000 deaths [3], with confirmed cases in all 26 states and in the Federal District.

In all countries, but especially in those of low and middle incomes, there is concern regarding the effects of the pandemic on the most impoverished populations [5, 6]. These population groups have difficulties in adopting preventive measures (such as social isolation); they are exposed to a context of pragmatic vulnerability that increases the risk of contamination, and, if infection occurs, they have limited access to health services. This is a complex, dynamic context that requires special attention from governments.

All of these conditions in which people live and which express, to a greater or lesser extent, the risk of illness, are called social determinants of health (SDH) [7]. The identification of the SDH that influence the dynamics of COVID-19 in Brazil is of fundamental importance for dealing with the pandemic and its consequences, thus contributing to the definition of mitigating public policies.

In Brazil, the Human Development Atlas and the Social Vulnerability Atlas are two important sources for studying the SDH, as they help to understand the context of the population’s living conditions and thus supporting decision making. These include the Municipal Human Development Index (MHDI) and the Social Vulnerability Index (SVI) with their respective dimensions. The MHDI considers development as “the capacity to expand the freedoms of individuals, in relation to their capacities and opportunities” [8]. Although advances have been observed, especially in the last three decades, the MHDI of 1/4 of Brazilian municipalities are considered low or very low. The SVI, on the other hand, measures “the access, absence, or insufficiency of some assets in areas of Brazilian territory, which should, in principle, be available to every citizen, due to the action of the State.” About 1/3 of the Brazilian municipalities are classified as having high or very high vulnerability [9].

The MHDI ranges from 0 to 1; the closer to 1, the greater the degree of human development in the municipality. Municipalities are classified into the following five development strata: very low (MHDI 0 to 0.499), low (MHDI 0.500 to 0.599), medium (MHDI 0.600 to 0.699), high (MHDI 0.700 to 0.799), and very high (MHDI 0.800 to 1) [8]. The SVI varies from 0 to 1; the closer to 1, the greater the degree of social vulnerability in the municipality. Municipalities are classified into the following five strata of vulnerability: very low (SVI 0 to 0.200), low (SVI 0.201 to 3.00), medium (SVI 0.301 to 0.400), high (SVI 0.401 to 0.500) and very High (SVI > 0.501) [9].

Based on this and on the need to understand the relationship between COVID-19 and the population’s living conditions, this study aimed to identify the SDH related to the incidence, mortality, and case fatality rates of COVID-19 in Brazil, in 2020.

## Methods

This is an ecological study involving all confirmed cases of COVID-19 in Brazil until May 6, 2020. In this study, the following three epidemiological indicators were adopted as dependent variables: i) COVID-19 incidence rate/100 000 inhabitants, ii) COVID-19 mortality rate/1 million inhabitants, and iii) COVID-19 case fatality rate (%). Data on cases and deaths were obtained from the CoVida network panel (https://painel.covid19br.org/), and population data were obtained from the Brazilian Institute of Geography and Statistics (IBGE, acronym in Portuguese) (https://www.ibge.gov. br /). The indicators were calculated, according to the following equations:

- COVID-19 incidence rate
  $$ \mathrm{COVID}-19\ \mathrm{incidence}\ \mathrm{Rate}=\frac{\mathrm{Number}\ \mathrm{of}\ \mathrm{cases}\ \mathrm{due}\ \mathrm{to}\ \mathrm{COVID}-19}{\mathrm{Resident}\ \mathrm{population}\ \mathrm{in}\ \mathrm{the}\ \mathrm{year}}\times 100\kern0.5em 000 $$

b) COVID-19 mortality rate
  $$ \mathrm{COVID}-19\ \mathrm{mortality}\ \mathrm{rate}=\frac{\mathrm{Number}\ \mathrm{of}\ \mathrm{deaths}\ \mathrm{due}\ \mathrm{to}\ \mathrm{COVID}-19}{\mathrm{Resident}\ \mathrm{population}\ \mathrm{in}\ \mathrm{the}\ \mathrm{year}}\times 1\ 000\ 000 $$

c) Case fatality rate
  $$ \mathrm{COVID}-19\ \mathrm{case}\ \mathrm{fatality}\ \mathrm{rate}=\frac{\mathrm{Number}\ \mathrm{of}\ \mathrm{deaths}\ \mathrm{due}\ \mathrm{to}\ \mathrm{COVID}-19\ }{\mathrm{Number}\ \mathrm{of}\ \mathrm{confirmed}\ \mathrm{cases}}\times 100 $$

The group of independent variables was composed of 49 indicators of human development and social vulnerability, obtained from the Municipal Human Development Atlas of MHDI (http://atlasbrasil.org.br/2013/) and the Social Vulnerability Atlas of SVI (http://ivs.ipea.gov.br/index.php/pt/).

The MHDI is composed of nine variables grouped into the following three categories:

- Longevity (one variable): life expectancy at birth;
- Education (seven variables): 1) sub-index of schooling, 2) percentage of individuals aged 18 years or over who have completed elementary school, 3) sub-index of school attendance, 4) percentage of individuals aged 5 to 6 years enrolled in school, 5) percentage of individuals aged 11 to 13 years who are enrolled in the final years of elementary school or who have completed elementary school, 6) percentage of individuals aged 15 to 17 years who have completed elementary school, and 7) percentage of individuals aged 18 to 20 years who have completed high school;
- Income (one variable): per capita income.

The SVI is composed of 16 variables grouped into the following three categories:

- Urban infrastructure (three variables): 1) percentage of people in households with inadequate water supply and sewage, 2) percentage of the population living in urban households without garbage collection service, and 3) percentage of people who live in households with per capita income less than half the minimum wage and who spend more than 1 h to reach their place of work out of the total number of employed, vulnerable people who return from work daily;
- Human Capital (eight variables): 1) mortality up to 1 year of age, 2) percentage of children from 0 to 5 years of age who do not attend school; 3) percentage of people aged six to 14 years who do not attend school; 4) percentage of women aged 10 to 17 years who have children; 5) percentage of mothers who are heads of household, without complete elementary school and with at least one child under the age of 15, out of the total number of mothers who are heads of household; 6) illiteracy rate of the population aged 15 years or over; 7) percentage of children living in households where none of the residents have completed elementary school; 8) percentage of people aged 15 to 24 years who do not study, do not work, and have a per capita household income equal to or less than half minimum wage (2010), out of the total population of this age group;
- Income and Work (five variables): 1) proportion of people with per capita household income equal to or less than half minimum wage (2010); 2) unemployment rate of the population aged 18 or over; 3) percentage of people aged 18 or over without complete elementary education and holding informal occupation; 4) percentage of people in households with per capita income below half minimum wage (2010) and dependent on the elderly; and 5) activity rate of people aged 10 to 14 years.

In addition to these, the following 16 variables that make up the Social Vulnerability Atlas and that express the population’s living conditions were included: 1) illiteracy rate of people 18 years or elder, 2) illiteracy rate of people 25 years or elder, 3) income per capita of those vulnerable to poverty, 4) percentage of income from work, 5) Gini Index, 6) percentage of employees 18 years or older with a formal contract, 7) percentage of employees 18 years or elder without a formal contract, 8) percentage of public sector workers 18 years or elder, 9) percentage of self-employed workers 18 years or elder, 10) percentage of employers 18 years or elder, 11) degree of formality of the employed 18 years or elder, 12) percentage of employed persons 18 years or elder who have completed primary education, 13) percentage of employed persons 18 years or elder who have completed secondary education, 14) percentage of employed persons 18 years or older who have completed higher education, 15) average income of employed persons 18 years or elder, and 16) percentage of employed persons 18 years or elder without income.

After data extraction, the variables were grouped into 10 blocks for statistical treatment. This organization aimed to reduce multicollinearity, which could compromise the quality of the study results. Data analysis was subsequently divided into the following four stages:

- **Step 1- Exploratory analysis of epidemiological indicators according to population size and human development and social vulnerability:** In this stage, municipalities were grouped according to population size and strata of human development and social vulnerability. Epidemiological indicators were subsequently calculated for each stratum, and exploratory analysis of rates was carried out.
- **Step 2- Analysis of bivariate spatial correlation:** Moran bivariate statistics and pseudo-significance test were used to assess the correlation between the incidence rate and the independent variables. The Moran index ranges from − 1 to + 1. Values close to zero indicating spatial randomness; positive values suggest positive spatial autocorrelation, and negative values suggest negative spatial autocorrelation [10]. Only variables with statistical significance (*P* <  0.05) in this stage were included in the next one. It should be noted that, in stages 2 and 3, only incidence rate was analyzed, given that only 821 municipalities (14.7%) had registered deaths on the date of collection.
- **Step 3 Multivariate analysis and spatial association:** The association between the COVID-19 incidence rate and the independent variables was initially tested with the use of classical multivariate regression (ordinary least squares [OLS]). The model residues were submitted to spatial dependence analysis by global Moran statistics to assess the need to incorporate a spatial component of the regression model, according to the decision model proposed by Luc Anselin [11, 12]. Once established, Lagrange multiplier tests were applied to define whether the most appropriate spatial model for the data set would be the spatial delay model (assigning an unknown value to the response variable Y) or the spatial error model (considering the spatial component as noise to be removed) [11]. Finally, residues from spatial models were subjected to Moran statistics again to verify spatial independence. In addition to this criterion, the following items were used to assess the quality of the final model: Akaike information criterion (AIC), Bayesian information criterion (BIC), log probability, and determination coefficient (R^2^). Analyses were performed using GeoDa software (version 1.10.0.8, University of Illinois, Urbana-Champaign, USA).

Because this study uses data in the public domain, Research Ethics Committee approval was waived.

## Results

### Spatial distribution

A total of 125 186 cases and 8452 deaths from COVID-19 were included in the study. Cases were reported in 2496 municipalities (44.8%), and deaths were reported in 821 municipalities (14.7%). Although the disease is present both in municipalities with large populations (> 100 000 inhabitants) and in those with small sizes (≤ 10 000), 81.3% of municipalities with up to 10 000 inhabitants have not yet registered cases of COVID-19. Municipalities with populations over 100 000 inhabitants were the first affected, and, to date, they have an incidence of 88.95/100 000 and a mortality rate of 61.36/1 million inhabitants. In second place are municipalities with up to 10 000 inhabitants, with incidence rate of 45.89/100000 and mortality rate of 27.26/1 million. It is noteworthy that the case fatality rate in municipalities with populations between 10 000 and 20 000 inhabitants is similar to that observed in large municipalities (6.81 and 6.90%, respectively) (Table 1).

**Table 1:** Incidence, mortality, and case fatality rate of COVID-19, according to population size, Municipal Human Development Index and Social Vulnerability Index. Brazil, 2020

Population size	No. of municipalities	No. of cases	No. of deaths	Resident population	Incidence rate/100 000	Mortality rate/1 million	CFR^1^ (%)
≤ 10 000 inhabitants	482	1296	77	2 824 212	45.89	27.26	5.94
10 001 to 20 000	580	2568	175	8 549 277	30.04	20.47	6.81
20 001 to 50 000	783	7075	390	24 491 464	28.89	15.92	5.51
50 001 to 100 000	328	6988	411	22 642 923	30.86	18.15	5.88
> 100 000	323	107 259	7399	120 576 849	88.95	61.36	6.90
**MHDI**^2^							
Very low (0–0.499)	18	289	9	489 795	59.00	36.75	3.11
Low (0.500–0.599)	588	4031	301	13 623 241	29.59	22.01	7.47
Medium (0.600–0.699)	830	13 021	832	31 335 083	41.55	26.55	6.39
High (0.700–0.799)	1.016	72 544	4891	100 556 389	72.14	48.63	6.74
Very high (0.800–1)	44	35 301	2419	33 080 217	106.71	73.12	6.85
**SVI**^3^							
Very low (0–0.200)	350	7428	362	20 782 329	35.74	17.42	4.87
Low (0.200–0.300)	703	54 736	3722	79 129 666	69.17	47.04	6.80
Medium (0.300–0.400)	485	49 528	3413	49 839 712	99.37	68.48	6.89
High (0.400–0.500)	534	8530	628	17 423 869	48.96	36.04	7.36
Very high (0.500–1)	424	4964	327	11 909 149	41.68	27.46	6.59
**Total**	**2496**	**125 186**	**8452**	**179 084 725**	**69.90**	**47.20**	**6.75**

^a^Data from May 6, 2020. ^1^*CFR* Case Fatality Rate, ^2^*MHDI* Municipal Human Development Index, ^3^*SVI* Social Vulnerability Index

### Human development and social vulnerability

In relation to human development, it was observed that all 44 municipalities with very high MHDI were affected by COVID-19. This group had the highest COVID-19 incidence rate (106.71/100 000) and mortality (73.12/1 million). In second place are municipalities with very low MHDI, 56.2% of which have already registered cases of the disease. Regarding incidence and COVID-19 mortality rate, the group with very low MHDI held third place (COVID-19 incidence rate: 59.00/100000 and COVID-19 mortality rate: 36.75/1 million) (Tables 1 and 2).

**Table 2:** Proportion of municipalities affected by COVID-19, according to population size, Municipal Human Development Index, and Social Vulnerability Index. Brazil, 2020

Population size	No. of municipalities	No. of municipalities with confirmed cases	% of municipalities with confirmed cases
≤ 10 000 inhabitants	2452	482	19.7
10 001 to 20 000	1344	580	43.2
20 001 to 50 000	1101	783	71.1
50 001 to 100 000	349	328	94.0
> 100 000	324	323	99.7
**MHDI**^1^			
Very low (0–0.499)	32	18	56.2
Low (0.500–0.599)	1367	588	43.0
Medium (0.600–0.699)	2233	830	37.2
High (0.700–0.799)	1889	1016	53.8
Very high (0.800–1)	44	44	100.0
**SVI**^2^			
Very low (0–0.200)	627	350	55.8
Low (0.200–0.300)	1699	703	41.4
Medium (0.300–0.400)	1258	485	38.5
High (0.400–0.500)	1178	534	45.3
Very high (0.500–1)	803	424	52.8

^a^Data from May 6, 2020. ^1^MHDI: Municipal Human Development Index, ^2^SVI: Social Vulnerability Index. The difference observed in the number of municipalities according to population size (5570) differs from the number of municipalities according to strata of MHDI and SVI (5565). This is due to the date of creation of new municipalities, after 2010

Regarding social vulnerability, 55.8% of the municipalities with very low SVI have already registered cases of COVID-19, followed by the municipalities with very high SVI (52.8%). Considering the incidence and mortality rates, municipalities with average SVI held first place (99.37 cases/100 000 and 68.48 deaths/1 000 000). It is also noteworthy that the municipalities with high SVI had higher case fatality rate (7.36%) (Tables 1 and 2).

### Association between living conditions and COVID-19

**Fig. 1:**
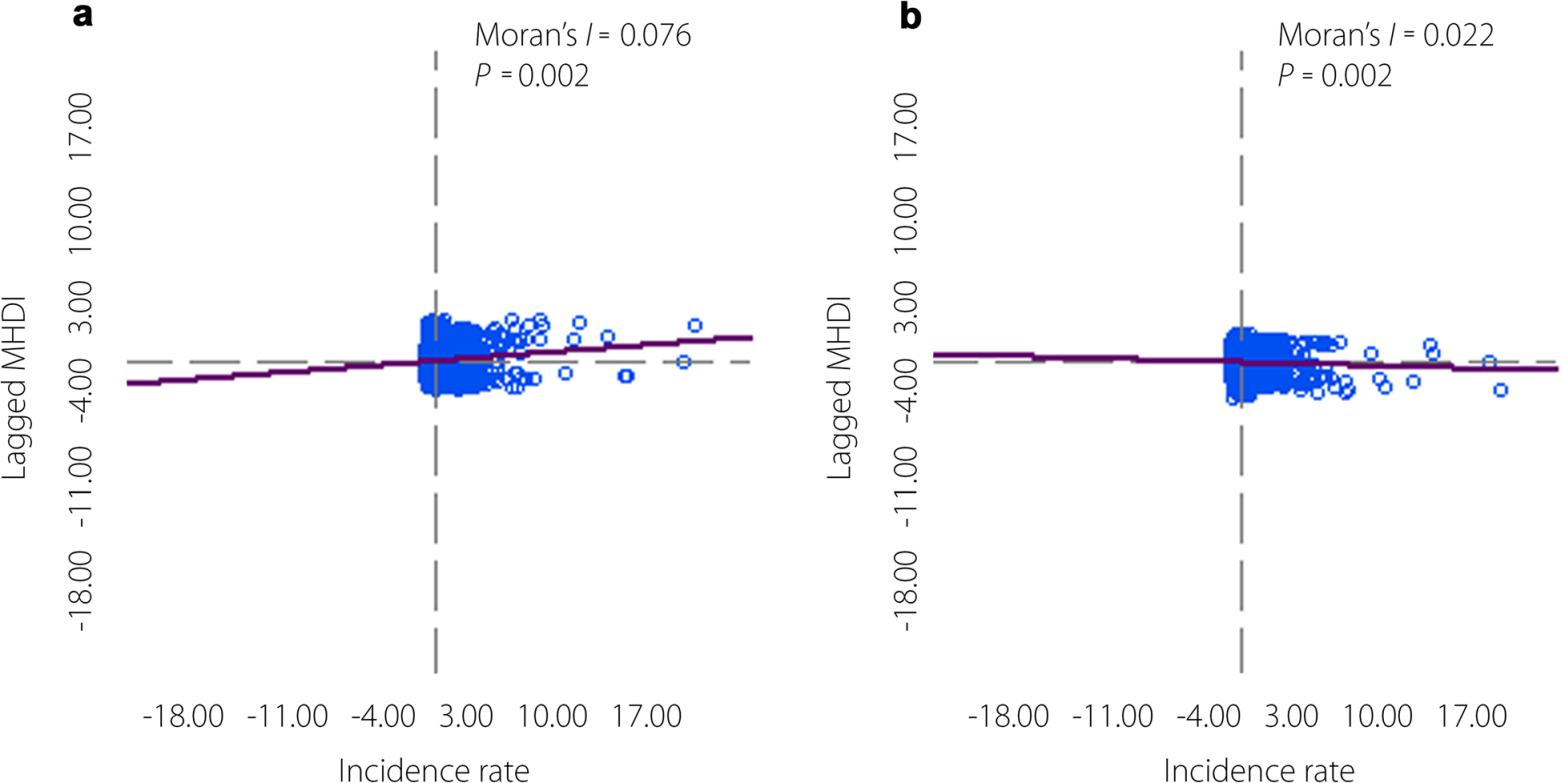
Moran’s bivariate spatial correlation between the incidence rate of COVID-19 in Brazil and the Social Vulnerability Index (SVI) and Municipal Human Development Index (MHDI). Brazil, 2020. a. Social vulnerability index; b. Municipal human development index

Initially, a positive bivariate spatial correlation was observed between the incidence rate of COVID-19 and the general SVI (Moran *I* = 0.076; *P* = 0.002) and a negative correlation with the MHDI (Moran *I* = -0.022; *P* = 0.002) (Fig. 1). Of the 49 variables analyzed, five showed no spatial correlation with the incidence rate of COVID-19 and were excluded from subsequent analyses. In the multivariate regression model, 21 variables were associated with incidence rate. None of the variables in block 2 (domains of the SVI) showed significance. In all other blocks of variables, the residuals of the regression model were spatially dependent. Lagrange multiplier tests indicated the spatial error model for block 8 (domains of the MHDI education) and spatial lag model for the others (Table 3).

**Table 3:** Moran bivariate correlation, multivariate regression, and spatial regression between COVID-19 incidence rate and social determinants of health. Brazil, 2020

Social determinants	Bivariate correlation		Multivariate regression		Spatial regression	
	Moran *I*	*P* value	Coefficient	*P* value	Coefficient	*P* value
**Block 1: synthetic indicators of social vulnerability and human development**						
Social Vulnerability Index	0.076	0.002	157.70	< 0.001	85.992	< 0.001
Municipal Human Development Index	-0.022	0.002	297.64	< 0.001	184.08	< 0.001
**Block 2: Domains of the Social Vulnerability Index**						
SVI urban infrastructure	0.012	0.024	-0.015	0.664	-	-
SVI human Capital	0.041	0.002	-14.374	0.445	-	-
SVI income and work	0.029	0.005	-0.150	1.000	-	-
**Block 3: Domains of the Municipal Human Development Index**						
MHDI longevity	-0.004	0.050	49.268	0.339	-2.2737	0.960
MHDI education	-0.018	0.002	71.587	0.002	60.566	0.004
MHDI income	-0.028	0.001	-65.9104	0.052	-26.068	0.394
**Block 4**: **SVI urban infrastructure domain**						
Percentage of people in households with inadequate water supply and sewage	0.111	0.002	0.298482	0.00323	0.0538755	0.55741
Percentage of the population living in urban households without garbage collection service	0.105	0.002	-0.105842	0.40675	-0.0830367	0.47279
Percentage of people who live in households with per capita income less than half minimum wage (2010) and who spend more than an hour to reach place of work	0.074	0.005	1.19532	0.00000	0.78499	0.00001
**Block 5: SVI human capital domain**						
Mortality up to 1 year old	-0.019	0.010	-0.0233645	0.94510	0.068086	0.82536
Percentage of children aged 0 to 5 who do not attend school	0.026	0.002	-0.480704	0.0050	-0.370537	0.0733
Percentage of people aged 6 to 14 who do not attend school	0.164	0.001	2.99476	0.00000	1.2672	0.00621
Percentage of women aged 10 to 17 who had children	0.0899	0.002	2.0033	0.01684	1.28813	0.09273
Percentage of mothers who are heads of household, without complete elementary school and with children under 15 years of age	0.0556	0.002	0.0249375	0.88425	-0.194968	0.21320
Illiteracy rate of the population aged 15 or over	-0.0456	0.004	-1.11608	0.00049	-0.66807	0.02257
Percentage of children living in households where none of the residents have completed elementary school	-0.0175	0.002	-0.372317	0.05105	-0.235047	0.17791
Percentage of people aged 15 to 24 who do not study, do not work and have a per capita household income equal to or less than half minimum wage (2010)	0.029	0.002	0.859119	0.00164	0.572682	0.02164
**Block 6: SVI income and labor domain**						
Proportion of people with per capita household income equal to or less than half minimum wage (2010)	0.0281	0.005	0.823252	0.00000	0.496891	0.00007
Unemployment rate of the population aged 18 or over	-0.0065	0.01	-0.643252	0.15191	-0.361881	0.37497
Percentage of persons aged 18 or over with no complete elementary education and informally employed	0.0048	0.002	-1.64513	0.00000	-1.05841	0.00000
Percentage of people in households with per capita income less than half the minimum wage (2010) and dependent on the elderly	-0.0031	0.198	-	-	-	-
Activity rate of persons aged 10 to 14 years of age	0.0943	0.005	1.31266	0.00000	0.586937	0.00466
**Block 7: MHDI longevity domain**						
Life expectancy at birth	-0.0044	0.030	0.986428	0.02657	0.89851	0.02545
**Block 8: MHDI education domain**						
Subindex education	0.0100	0.002	2728.69	0.52713	294.603	0.93733
Percentage of individuals aged 18 or older who have completed elementary school	0.0100	0.002	-26.5486	0.53830	-2.21157	0.93733
School attendance index	-0.0430	0.002	74.3527	0.32880	-40.6921	0.56735
Percentage of individuals aged 5 to 6 years in school	-0.0731	0.002	-0.55266	0.03744	-0.017439	0.94772
Percentage of individuals aged 11 to 13 years old who are enrolled in the final years of elementary school or who have completed elementary school	-0.0775	0.005	-0.536377	0.08705	0.644394	0.03203
Percentage individuals aged 15 to 17 years, with complete primary	-0.0226	0.002	-0.405591	0.21912	0.0235485	0.93707
Percentage individuals aged 18 to 20 years who have graduated from secondary school	-0.005	0.382	-	-	-	-
**Block 9: MHDI income domain**						
Per capita income	-0.0113	0.002	0.228899	0.00799	0.199184	0.01070
**Block 10: Other vulnerability and development indicators**						
Illiteracy rate (aged 18 years or over)	-0.037	0.062	-	-	-	-
Illiteracy rate (aged 25 years or over)	-0.035	0.064	-	-	-	-
Per capita income of those vulnerable to poverty	-0.060	0.002	-0.109244	0.00003	0.00382703	0.96425
Percentage of income from income from work	0.074	0.001	0.869056	0.19392	0.309083	0.10397
Gini Index	0.049	0.170	-	-	-	-
Percentage of employees with a formal contract (aged 18 years or over)	-0.055	0.002	-0.622194	0.19392	-0.533661	0.22342
Percentage of employees without a formal contract (aged 18 years or over)	0.039	0.4060	-	-	-	-
Percentage of public sector workers (aged 18 years or over)	0.0286	0.002	0.457244	0.45981	0.566954	0.31684
Percentage of self-employed workers (aged 18 years or over)	0.1020	0.001	0.386326	0.12980	-0.092865	0.69078
Percentage of employers (aged 18 years or over)	-0.0292	0.005	-5.14761	0.00078	-4.63911	0.00094
Degree of formality of employed persons (aged 18 years or over)	-0.0424	0.005	0.102908	0.79265	-0.0738698	0.83667
Percentage of employed persons with complete elementary education (aged 18 years or over)	-0.0039	0.005	0.99806	0.03399	0.889692	0.03927
Percentage of employed persons with complete high school (aged 18 years or over)	-0.0074	0.002	0.71983	0.20002	0.189241	0.71284
Percentage of employed persons with complete higher education (aged 18 years or over)	-0.0378	0.002	-2.44724	0.00030	-1.59828	0.00990
Average income of employed persons (aged 18 years or over)	-0.0036	0.002	0.0317216	0.00145	0.0317537	0.00050
Percentage of employed persons without income (aged 18 years or over)	0.0422	0.001	0.890013	0.00021	0.373849	0.08911

*MHDI* Municipal Human Development Index, *SVI* Social Vulnerability Index. - Not applicable

The spatial regression model, finally, identified 17 indicators associated with incidence rate, 13 of which showed positive association, namely the following: SVI; MHDI; MHDI education; Percentage of people who live in households with per capita income less than half minimum wage (2010) and who spend more than 1 h to reach place of work; percentage of people aged six to 14 years who do not attend school; percentage of people aged 15 to 24 years who do not study, do not work, and have per capita household income equal to or less than half the minimum wage (2010); percentage of people with per capita household income equal to or less than half minimum wage (2010); activity rate of people aged 10 to 14 years; life expectancy at birth; percentage of individuals aged 11 to 13 who are enrolled in the final years of elementary school or who have completed elementary school; per capita income; percentage of employed persons aged 18 or elder with complete elementary school; and average income of employed persons aged 18 or over. Four variables showed a negative association, namely, illiteracy rate of the population aged 15 years or over; percentage of employers aged 18 or over; percentage of people aged 18 or over without complete elementary education holding informal occupation; and percentage of employed people aged 18 or over with complete higher education (Table 3).

## Discussion

COVID-19 currently represents the main global health, social, and economic challenge. In Brazil, the spread of the disease started in the most developed municipalities in the country, and it has spread throughout the Brazilian territory without delay, reaching smaller and more vulnerable areas whose populations are exposed to a chronic and historical context of social deprivation. This process of spatial dissemination justifies the complex influence of SDH on the spread of the virus across the country.

Considering that the virus is reaching the most vulnerable and least developed municipalities in the country after those with better living conditions, there is reason to believe that these municipalities will be more severely affected and will suffer incalculable consequences, if consistent support measures are not adopted urgently.

The first reason concerns the risk context of these populations and the difficulties in implementing and/or adopting preventive measures. Approximately 37 million Brazilian workers earn their income from activities related to the sale of products and the provision of services, constituting a population vulnerable to contamination [13]. In poorer municipalities, the percentage of these populations rises considerably [13]. Furthermore, in many cases, this is the only source of income for the family’s subsistence, which makes adherence to voluntary social isolation difficult to maintain for a long period without the proper support of emergency public policies.

COVID-19, therefore, has a double effect on the most vulnerable populations, to the extent that it both perpetuates poverty and is perpetuated by poverty itself. It is perpetuating in the sense that, for each percentage point reduction in the global economy, it is estimated that an additional 10 million people will be placed in poverty [5]. In Brazil, the pandemic could increase by 6.5 percentage points in the poverty index, thus affecting almost one quarter of the Brazilian population [14]. It would furthermore increase inequality in income distribution (6.5% increase in the Gini Index) [14]. In the states of the North and Northeast Regions, these effects of pandemics can be even more pronounced, given that they are the least developed regions with the most vulnerable populations.

It is perpetuated by poverty, because this vulnerable population, having no financial reserves and depending on emergency government assistance, will scarcely be able to adhere to non-pharmacological preventive measures, such as social isolation, wearing masks, and hand hygiene. In this regard, living conditions are able to maintain the COVID-19 transmission chain active. In our study, there was a higher incidence rate in municipalities with greater social vulnerability; higher proportion of people who live in households with per capita income less than half minimum wage and who spend more than 1 h to reach place of work; higher proportion of children aged six to 14 years who do not attend school; and higher proportion of people aged 15 to 24 years who do not study, do not work and have a per capita income of less than half minimum wage. These populations are more likely not to follow government recommendations [15].

The lack of home structure and the lack of access to minimum resources, such as water and basic sanitation, both on the outskirts of large cities and in municipalities in the interior of the country, can increase the risk of illness due to COVID-19, as observed with other respiratory diseases [16]. In Brazil, four million families do not have a bathroom at home; 35 million do not yet have access to treated water, and 100 million do not have a sewage network [17]. Therefore, it is possible to state that the degree of suffering generated by the pandemic depends on the area where individuals live and the social conditions to which they are subjected [18].

Even in rich countries, such as the United States of America (USA), the social inequalities that exist in cities determine the greater or lesser risk of illness for their residents. In Boston, for example, there is a high concentration of poverty and a prevalence of diseases caused by it in certain areas and good living conditions and a low prevalence of these diseases in other nearby points [19, 20]. This scenario reinforces the relationship between social policies and the health conditions experienced by the population.

A study carried out in the USA, with data from 433 cities, involving 283 256 cases and 6644 deaths from COVID-19, showed that the highest social vulnerability index was associated with a higher incidence and lethality due to the disease (Relative Risk- RR = 1.19), being still higher when considering the population aged 65 or older (RR = 1.63). In the study, 28.9% of the municipalities had a high social vulnerability (SVI ≥ 0.46) and a high adjusted lethality rate (≥ 2.3%) [21].

In addition, mortality caused by the disease, especially in household providers, can increase the poverty of families. In this investigation, municipalities with small population sizes, as well as those with low MHDI and high SVI already show high mortality rates. The situation tends to become more critical when all municipalities are affected, which will not take long to happen. Even with the set of actions implemented by the Brazilian government, through “Brazil’s response policy to COVID-19” [22], it is likely that after this pandemic, Brazil will face a second crisis related to poverty and the diseases associated therewith. The loss of income can bring other consequences for the health of the population, such as a higher frequency of mental illnesses, increased consumption of alcohol and other drugs and increased domestic violence [23]. It is not yet possible to predict the size of the impact of the pandemic on people’s lives, especially on the most vulnerable and, for this reason, strategies to combat COVID-19 must be developed on different fronts of action.

The second reason refers to the capacity of municipalities to face the contamination of their population and to offer conditions for detection and treatment of patients. If we consider that 7% of those infected will need hospitalization and that 2% will require intensive care [24], the group of municipalities with very high vulnerability (which recorded 4964 cases of COVID-19) would need 347 hospital beds and 99 intensive care unit (ICU) beds.

Several factors make this scenario even more worrying. First, these small and more vulnerable municipalities do not have a hospital structure or ICU beds to meet the demand imposed by the pandemic [25]. In general, these beds are concentrated in municipalities that are regional health centers that serve the municipalities through agreements between managers. Second, because the disease first arrived in the larger municipalities with higher human development, these beds are already occupied. Third, these populations depend exclusively on beds in the public Unified Health System (SUS, acronym in Portuguese). About 94.4% of the individuals who constitute poorest 20% of the population are dependent on the SUS [21]. For this reason, the strengthening of all components of the health system is of fundamental importance for tackling the pandemic in Brazil [26, 27].

Even with all the precautions adopted, this study has some limitations, among which the following stand out: i. the underreporting of COVID-19 cases due to the limited availability of tests and the capacity of local surveillance services, ii. deficiencies in investigating deaths due to the disease, with significant underreporting, and iii. Use of secondary data that are subject to constant variation.

## Conclusions

Concerns with the advance of the COVID-19 pandemic in the country’s smallest, most vulnerable, and least developed municipalities raise the alert for Brazilian political authorities. It is necessary to do the following: 1) delay the arrival of the disease in these locations by adopting effective prevention mechanisms that consider the collective risk of illness, the social context experienced by these populations, and the best existing scientific evidence; 2) expand and prepare the health network with urgent investments in the SUS at all levels of care; and 3) guarantee social protection for the vulnerable population.

## Data Availability

Not applicable.
